# Physiological activity in calm thermal indoor environments

**DOI:** 10.1038/s41598-017-11755-3

**Published:** 2017-09-14

**Authors:** Tsuyoshi Okamoto, Kaori Tamura, Naoyuki Miyamoto, Shogo Tanaka, Takaharu Futaeda

**Affiliations:** 1 Faculty of Medical Sciences, Kyushu University, 3-1-1 Maidashi, Higashi-ku, Fukuoka, 812-8582 Japan; 2 Faculty of Arts and Science, Kyushu University, 744 Motooka, Nishi-ku, Fukuoka, 819-0395 Japan; 3 Graduate School of Systems Life Sciences, Kyushu University, 744 Motooka, Nishi-ku, Fukuoka, 819-0395 Japan; 4 Anny Group, 6-3 Tenya-machi, Hakata-ku, Fukuoka, 812-0025 Japan

## Abstract

Indoor environmental comfort has previously been quantified based on the subjective assessment of thermal physical parameters, such as temperature, humidity, and airflow velocity. However, the relationship of these parameters to brain activity remains poorly understood. The objective of this study was to determine the effect of airflow on brain activity using electroencephalograms (EEG) of participants in a living environment under different airflow conditions. Before the recording, the room was set to a standardised air temperature and humidity. During the recording, each participant was required to perform a simple time-perception task that involved pressing buttons after estimating a 10-second interval. Cooling and heating experiments were conducted in summer and winter, respectively. A frequency analysis of the EEGs revealed that gamma and beta activities showed lower amplitudes under conditions without airflow than with airflow, regardless of the season (*i.e*., cooling or heating). Our results reveal new neurophysiological markers of the response to airflow sensation. Further, based on the literature linking gamma and beta waves to less anxious states in calm environments, we suggest that airflow may alter the feelings of the participants.

## Introduction

A comfortable indoor thermal environment can improve the quality of sleep^1^ and work productivity^2–4^. Previous studies have generated evaluation scales for indoor thermal comfort based on physical and personal factors^5–9^, with representative physical factors including room temperature, humidity, and airflow velocity^5^. An international common scale, the Predicted Mean Vote (PMV)^6^, has been established to summarise and standardise these factors, and it has been widely accepted in many countries^5, 7^. The PMV and other similar scales (*e.g*., the Standard New Effective Temperature)^8, 9^ are based on results from an extensive survey of subjective evaluations.

Comfort is a highly subjective feeling and cannot be measured in an objective manner in principle. Although PMV is widely used as a reliable scale, it has not yet been able to fully elucidate the mechanisms underlying this feeling. More specifically, the brain mechanism that perceives and processes the environmental state evaluated by PMV is unclear. The relationship between PMV and neuronal responses should be investigated in order to better understand the mechanisms underlying comfort.

Airflow is one of the factors used to calculate PMV, and its sensation and underlying neuronal mechanisms have been investigated by a number of researchers. Although many investigations have shown the unpleasant effects of air velocity and draught by subjective reactions^10–15^, few studies have addressed neurophysiological mechanisms underlying unpleasantness induced by airflow. In addition to using thermal scales, measuring neurophysiological activity could help to determine the brain-intrinsic factors required to understand the mechanism underlying feelings induced by calm environments.

In this study, our aim was to determine the effects of airflow sensation in indoor thermal environments by performing electroencephalography (EEG). Neurophysiological studies conducted in experimental laboratories have reported that the amplitudes of gamma and beta oscillations increase during vigilance states (*i.e*., sustained attention)^16, 17^ or mental fatigue^18, 19^. The neuronal mechanisms of airflow sensation may be revealed by analysing these types of oscillation changes using EEG measurements.

**Figure 1:**
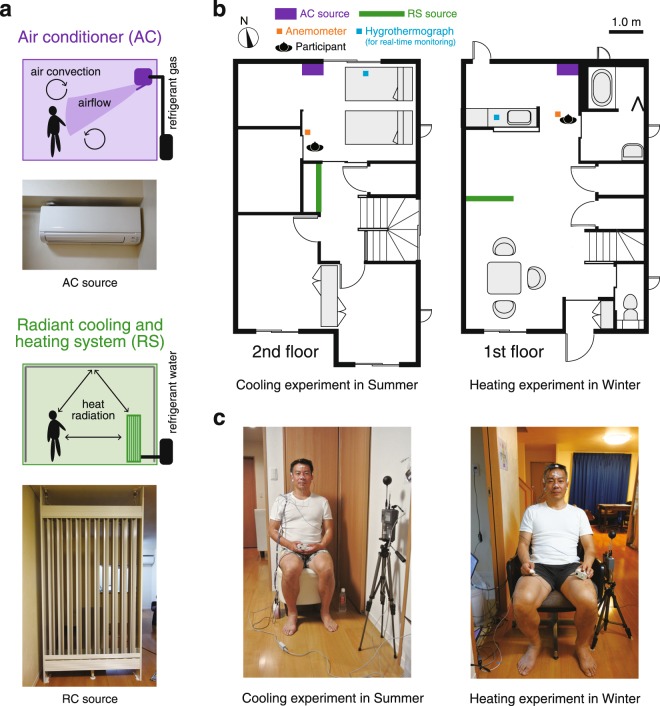
Experimental environment. (**a**) System summary of the air conditioner and radiant cooling and heating system. (**b**) Floor plans of the rooms used for our experiments. (**c**) A participant in each experiment. The left figure shows the conditions for the cooling experiment; the right figure, the conditions for the heating experiment.

**Figure 2:**
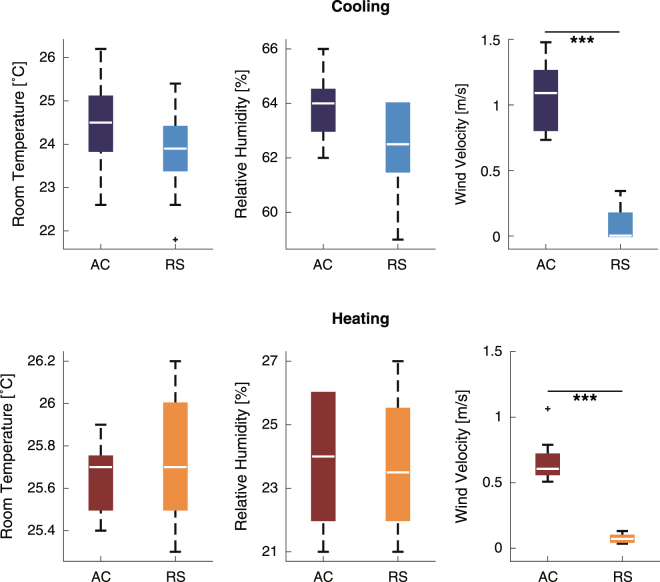
The room temperature, relative humidity, and wind velocity of the cooling experiment (upper panels) and the heating experiment (lower panels). The asterisks showed the significant differences (***p < 0.001).

In order to examine the effects of airflow in indoor environments that mimic daily life, we conducted experiments under two different seasonal conditions (cooling in summer and heating in winter) and two different airflow conditions (an air conditioner with airflow and a radiant cooling and heating system without airflow) in an environment standardised for temperature and humidity (see Methods and Figs 1 and 2). To standardise the mental states of the participants, we introduced a time-counting task. In each session, the participants were asked to press a button after mentally counting for 10 seconds with their eyes closed. This was repeated for up to 60 seconds per session. EEGs were recorded during five sessions, and frequency analysis was carried out.

We hypothesised that changes in brain activity occur in response to different airflow conditions. Our aim was to determine neurophysiological markers of airflow sensation under conditions of both cooling and heating.

## Results

### Common scales for thermal comfort

Room temperature and relative humidity were monitored and maintained at the same levels by an experimenter. For confirmation of the condition, we performed statistical analyses of the data measured at the beginning of the experiments. There was no significant difference in room temperature between the air-conditioned (AC) environment and the radiant cooling and heating system (RS) environment (cooling: Z = −1.4, p = 0.16, heating: Z = 0.67, p = 0.50 by Wilcoxon test) (Fig. 2, left panels), and no significant differences in the relative humidity (cooling: Z = −1.9, p = 0.056, heating: Z = −0.088, p = 0.93) (Fig. 2, middle panels).

We also compared air velocities in the experiment room (Fig. 2, right panels). There were significant differences in air velocity between the AC and RS conditions. The median (interquartile range) of air velocity in the cooling experiment was 1.1 (0.46) m/s in the AC condition and 0.0033 (0.22) m/s in the RS condition (Z = −4.2, p < 0.001, Wilcoxon test) (Fig. 2, upper right). The median air velocity in the heating experiment was 0.61 (0.18) m/s in the AC condition and 0.071 (0.053) m/s in the RS condition (Z = −4.1, p < 0.001) (Fig. 2, lower right).

**Figure 3:**
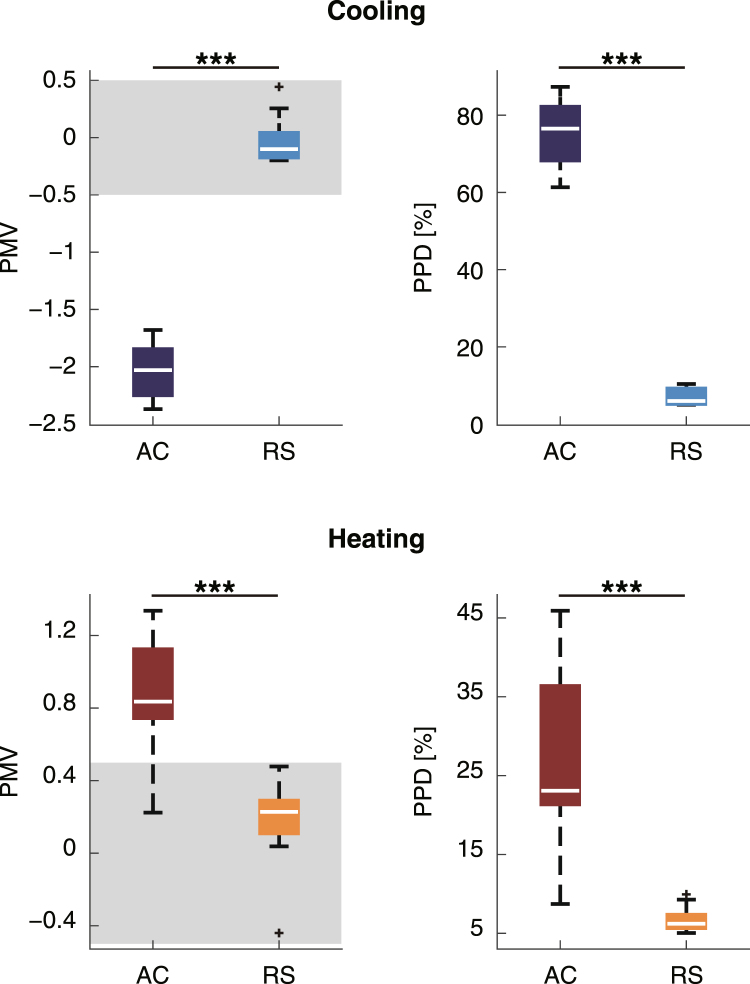
PMV and PPD values obtained by collapsing across the sessions in the cooling experiment (upper panels) and the heating experiment (lower panels). The asterisks showed the significant differences (***p < 0.001). The grey areas in the left panels indicate the thermal comfort zone defined by the ASHARE Standard.

To compare the EEG results with subjective ratings of thermal comfort and sensation, we used two thermal scales: the PMV and the Predicted Percentage of Dissatisfied (PPD) scales^5, 7^ (Fig. 3). The PMV is used to express thermal comfort as a sum of scores based on several types of environmental variables, the participant’s metabolic rate, and the level of clothing insulation^6^. The seven-point American Society of Heating, Refrigerating, and Air-Conditioning Engineers (ASHARE) thermal sensation scale is defined by the PMV (−3: cold, −2: cool, −1: slightly cool, 0: neutral, +1: slightly warm, +2: warm, and +3: hot). The thermal comfort zone indicated by the ASHARE Standard 55^5, 7^ ranges from −0.5 to +0.5.

In the cooling experiment, the median (interquartile range) of the PMV in the AC (with airflow) condition was −2.0 (0.44) and that in the RS (without airflow) condition was −0.10 (0.29). These values were significantly different (Z = 3.5, p < 0.001, Wilcoxon test) (Fig. 3, upper left). PPD, which measures the ratio of the number of people who feel dissatisfied to a parent population, was also significantly different in the conditions with and without airflow (median (interquartile range)﻿) of PPD: 6.0 (4.2)% in RS, 77 (15)% in AC, Z = −3.5, p < 0.001) (Fig. 3, upper right). In the heating experiment, the median PMV in the RS condition was significantly different from that in the AC condition (0.23 (0.23) and 0.84 (0.43), respectively; Z = −3.8, p < 0.001) (Fig. 3, lower left). PPD was also significantly different in the two airflow conditions (median (interquartile range﻿))in AC = 23 (17)%, median (interquartile range) in RS = 6.2 (1.8)%; Z = −4.0, p < 0.001) (Fig. 3, lower right). The median PMV scores and their interquartile ranges in the RS condition were within the range of comfort (from −0.5 to 0.5) in both the cooling and heating experiments.

### Neurophysiological activity

To compare the conditions with and without airflow, changes in EEG amplitudes in each session relative to the first session were calculated for each frequency band. These bands comprised theta (4–8 Hz), alpha (8–14 Hz), beta (14–30 Hz), and gamma (30–55 Hz) oscillations. The locations of the recording electrodes were chosen to enable measurement of responses based on both mental state and sensation (see Methods, EEG analysis).

**Figure 4:**
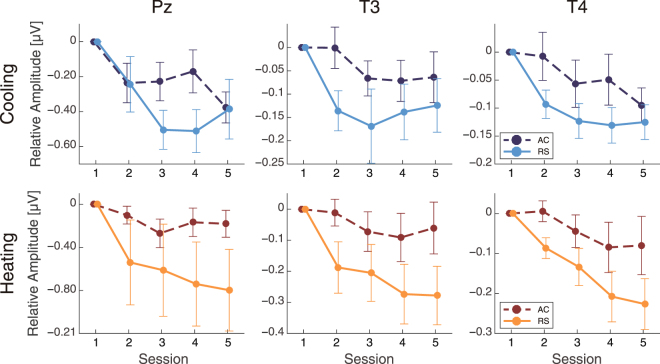
The differential gamma amplitudes (mean ± SEM) from the first session.

Gamma band amplitudes at temporal and parietal sites (Pz, T3, and T4) had smaller relative changes in the RS condition than in the AC condition in both experiments (Fig. 4). A two-way analysis of variance (ANOVA) indicated a significant main effect of airflow on gamma band amplitudes at each focal electrode in each experiment (cooling, Pz: F(1,72) = 4.4, p = 0.040, T3: F(1,72) = 8.8, p = 0.0040, T4: F(1,72) = 9.1, p = 0.0036; heating, Pz: F(1,72) = 11, p = 0.0016, T3: F(1,72) = 20, p < 0.001, T4: F(1,72) = 16, p < 0.001). A main effect of session was also significant or marginally significant (cooling, Pz: F(4,72) = 5.4, p < 0.001, T3: F(4,72) = 2.9, p = 0.028, T4: F(4,72) = 5.0, p = 0.0013; heating, Pz: F(4,72) = 2.2, p = 0.076, T3: F(4,72) = 4.2, p = 0.0040, T4: F(4,72) = 7.1, p < 0.001). There was no significant interaction between airflow condition and sessions (cooling, Pz: F(4,72) = 1.5, p = 0.20, T3: F(4,72) = 0.85, p = 0.50, T4: F(4,72) = 0.88, p = 0.48; heating, Pz: F(4,72) = 0.83, p = 0.51, T3: F(4,72) = 1.4, p = 0.24, T4: F(4,72) = 1.3, p = 0.30). We confirmed that the PMV value and gamma amplitudes were correlated in both experiments (cooling: r = −0.24, p = 0.031, heating: r = 0.36, p = 0.0031 at T3) (see Figs S1a and S2a in Supplementary Information). Analysis of gamma and PPD also confirmed a marginally significant correlation with cooling (r = 0.21, p = 0.065) and a significant correlation with heating (r = 0.31, p = 0.013 at T3) (Figs S3a and S4a in Supplementary Information). Although these results supported the relationships between gamma and PMV or PPD, the analytic data were measured repeatedly. To solve this problem, we analysed the correlations using data averaged across sessions. However, we could not find any significant correlations. This may be because of the small degree of freedom (Figs S5a–S8a and Tables S1–S4 in Supplementary Information). We introduced a new index of “two-point slope” to investigate the relationship between EEG amplitudes and PMV or PPD in each session (see Supplementary Methods). Median values of the two-point slopes in gamma that were significantly different from zero potentially indicated the presence of some relationship above chance level between PPD and EEG as independent measurements. Two-point slopes calculated from gamma and PMV had significant differences from zero at session 2 and a marginally significant difference at session 3 in cooling (session 2: Z = −16, p = 0.037; session 3: Z = −14, p = 0.065; Fig. S9a and Table S5 in Supplementary Information), as determined using a one-sample Wilcoxon test (one-sided). In heating, there were significant differences from zero at session 4 and marginal significances at sessions 2 and 5 (session 2: Z = 12, p = 0.055; session 4: Z = 14, p = 0.027; session 5, Z = 12, p = 0.055; Fig. S11a and Table S7 in Supplementary Information). We also assessed the two-point slopes between gamma and PPD and found significant differences and marginal significances in cooling (session 2: Z = 16, p = 0.037; session 3: Z = 14, p = 0.065; Fig. S10a and Table S6 in Supplementary Information) and heating (session 2: Z = 12, p = 0.055; session 3: Z = 10, p = 0.098; session 4: Z = 14, p = 0.027; session 5: Z = 12, p = 0.055; Fig. S12a and Table S8 in Supplementary Information). These results indicate that the higher gamma amplitudes may be candidate factors for explaining the subjective evaluations made using the PMV and PPD.

**Figure 5:**
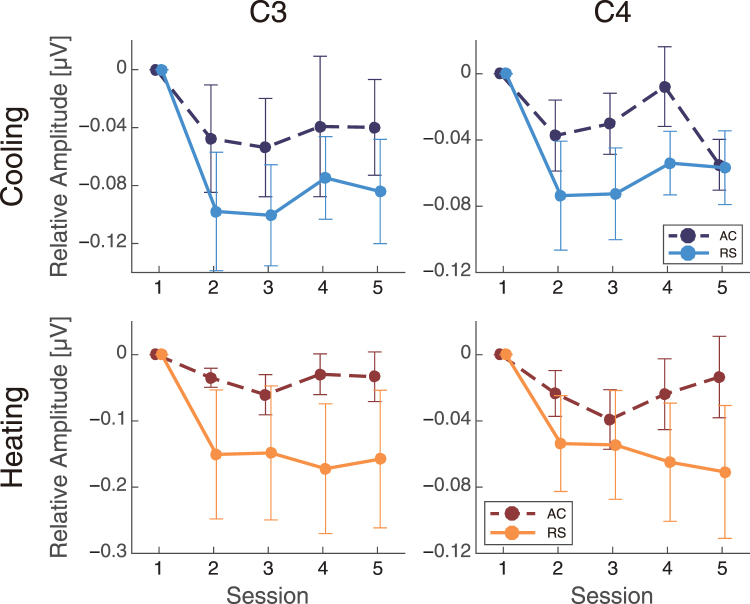
The differential beta amplitudes (mean ± SEM) from the first session.

Beta band amplitudes at central sites (C3 and C4) also had smaller relative changes in the RS condition than in the AC condition in both heating and cooling experiments (Fig. 5). An ANOVA indicated a significant main effect of airflow on beta band amplitudes (cooling, C3: F(1,72) = 5.0, p = 0.030, C4: F(1,72) = 6.0, p = 0.017; heating, C3: F(1,72) = 10, p = 0.023, C4: F(1,72) = 8.5, p = 0.0048). Main effects of session were also found at both C3 and C4 in the cooling experiment (C3: F(4,72) = 3.1, p = 0.022, C4: F(4,72) = 4.3, p = 0.0036) and at the C4 site in the heating experiment (C3: F(4,72) = 1.8, p = 0.15, C4: F(4,72) = 3.1, p = 0.021). However, there was no significant interaction between the airflow condition and session (cooling, C3: F(4,72) = 0.33, p = 0.86, C4: F(4,72) = 0.95, p = 0.44; heating, C3: F(4,72) = 0.71, p = 0.59, C4: F(4,72) = 1.0, p = 0.41). Correlation analysis between PMV and beta amplitudes showed a significant correlation in the cooling (r = −0.23, p = 0.041 at C3) (see Fig. S1b in Supplementary Information) and a marginal significant correlation in the heating experiment (r = 0.23, p = 0.062 at C3) (see Fig. S2b in Supplementary Information). Analyses of beta and PPD also confirmed a significant correlation in cooling (r = 0.22, p = 0.049 at C3) but not in heating (r = 0.15, p = 0.24 at C3) (Figs S3b and S4b in Supplementary Information). These results suggest that the beta activity is somewhat correlated with subjective feelings in the cooling state, while there is no correlation in the heating state. We also performed analyses of two-point slopes, as we did for gamma activity, to investigate the relationship between PMV or PPD and beta more deeply (Supplementary Methods). The two-point slopes of PMV did not have significant differences with zero in any of the sessions in the cooling state (p ≥ 0.10, Fig. S9b and Table S5 in Supplementary Information), although there was a marginally significant difference at session 5 in heating (session 5: Z = 10, p = 0.098, Fig. S11b and Table S7 in Supplementary Information), as assessed using a one-sample Wilcoxon test (one-sided). In addition, the two-point slopes of PPD did not have significant differences in cooling and heating (P ≥ 0.10, Fig. S10b and Table S6 in Supplementary Information), although there was a marginal difference at session 5 in heating (session 5: Z = 10, p = 0.098, Fig. S12b and Table S8 in Supplementary Information). Analyses of the two-point slopes provided us with no evidence to support a relationship between beta and thermal scales, PMV, or PPD.

**Figure 6:**
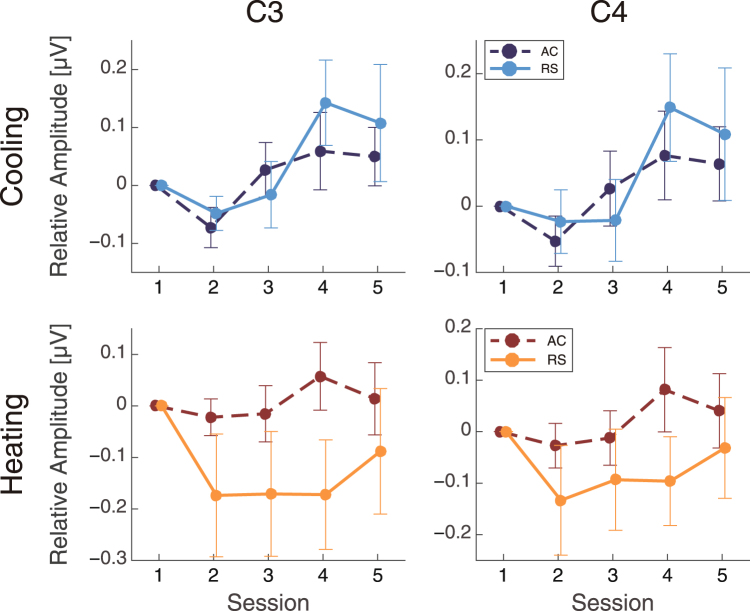
The differential alpha amplitudes (mean ± SEM) from the first session.

Alpha band amplitudes had different patterns in the cooling and heating experiments (Fig. 6). In the cooling experiment, the relative changes in the alpha band did not indicate a main effect of airflow (C3: F(1,72) = 0.67, p = 0.42, C4: F(1,72) = 0.42, p = 0.52). However, a main effect of airflow was observed in the heating experiment (C3: F(1,72) = 13, p < 0.001, C4: F(1,72) = 7.4, p = 0.0080). In addition, there was a main effect of session in the cooling experiment (C3: F(4,72) = 3.8, p = 0.0079, C4: F(4,72) = 3.5, p = 0.011), but not in the heating experiment (C3: F(4,72) = 1.0, p = 0.40, C4: F(4,72) = 1.1, p = 0.37). No interactions between these factors were found (cooling, C3: F(4,72) = 0.54, p = 0.71, C4: F(4,72) = 0.46, p = 0.76; heating, C3: F(4,72) = 1.1, p = 0.36, C4: F(4,72) = 0.80, p = 0.53).

Theta band amplitudes were also analysed, but no main effect of airflow was found (cooling, Fz: F(1,72) = 1.3, p = 0.25; heating, Fz: F(1,72) = 0.081, p = 0.15). A main effect of session was found in both heating and cooling experiments (cooling, Fz: F(4,72) = 9.3, p < 0.001, heating, Fz: F(4,72) = 3.2, p = 0.019), but no significant interaction between airflow and session was found (cooling, Fz: F(4,72) = 0.20, p = 0.93, heating, Fz: F(4,72) = 0.29, p = 0.89).

In brief, gamma and beta amplitudes indicated main effects in the airflow conditions in both cooling and heating environments. The gamma and beta activities may therefore reflect the sensation of airflow itself because differences were observed in both experiments regardless of the season. In contrast, alpha amplitudes indicated a main effect of airflow only in the heating experiment. Therefore, the pattern of alpha activity may be affected by differences in thermal sensation. Theta band amplitudes did not indicate any main effects of airflow conditions at the electrode locations of interest.

### Time-precision task

**Figure 7:**
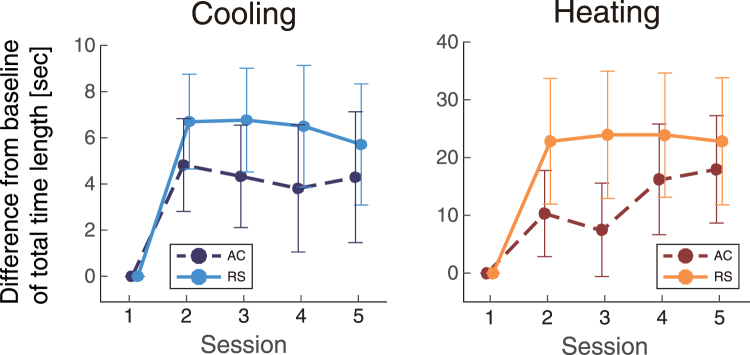
The differential total time lengths (TTLs, mean ± SEM) from the first session.

We analysed the total time length (TTL, approximately 60 seconds) of the task period for each participant. The TTL in the RS condition was longer than that in the AC environment in both the cooling and heating experiments (Fig. 7). A main effect of airflow was detected on TTL in both cooling and heating environments (cooling: F(1,70) = 10, p = 0.0027, heating: F(1,56) = 4.4, p = 0.040). These results indicate that the participants estimated time to be longer in RS environments, which lacked airflow, in both experiments. There was no main effect of session on TTL (cooling: F(3,70) = 0.27, p = 0.85, heating: F(3,56) = 0.23, p = 0.87).

### Thermography data of skin temperature

**Figure 8:**
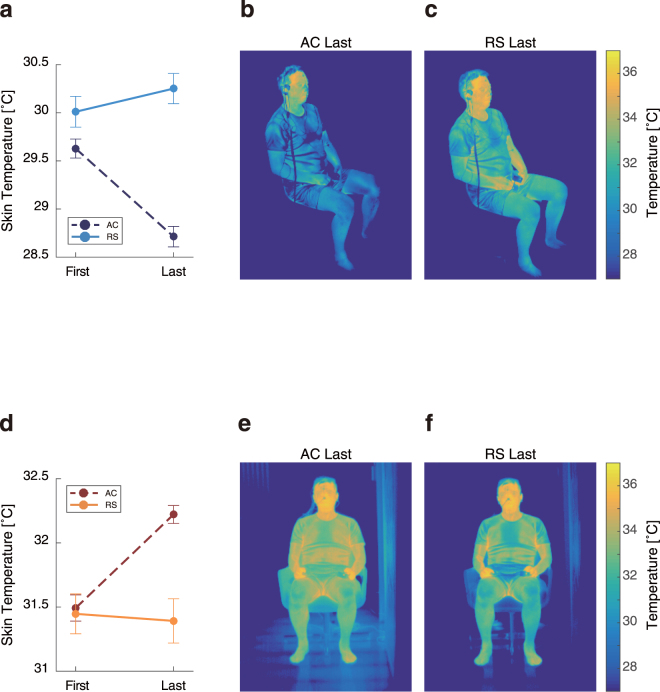
Skin temperatures (mean ± SEM) in the cooling experiment (**a**) and the heating experiment (**d**), and examples of thermography during the cooling (**b**, **c**) and heating (**e**, **f**) experiments.

Airflow has been found to directly influence skin temperature^15^. At the beginning and end of each experiment, the skin temperature of each participant was measured by using thermography (Fig. 8).

In the cooling experiment, the measured skin temperatures in the AC condition at the end of the experiment were lower than those at the beginning (t(22) = −6.3, p < 0.001, two-tailed Student’s *t*-test). In contrast, skin temperatures in the RS condition were not different between the first and last sessions (t(22) = 1.1, p = 0.30). In the heating experiment, the measured skin temperatures in the AC condition at the last session was higher than those at the first session (t(16) = 5.9, p < 0.001). In contrast, skin temperatures in the RS condition were not different between the first and last sessions (t(16) = −0.24, p = 0.59). These results confirm that the airflow condition only influences body surface temperatures.

## Discussion

We investigated the effects of airflow on brain activity in a living environment. We compared conditions with and without airflow using neurophysiological responses. As expected, the PMV, PPD, and task performance indicated higher subjective comfort in the environment without airflow. Furthermore, several EEG frequency bands showed significant differences between these conditions, even at the same room temperature. These results suggest that the observed neuronal responses are related to airflow in an indoor environment. Here we characterise the properties of indoor airflow sensation, as determined by brain-intrinsic factors underlying feelings associated with calm environments. To our knowledge, this is the first report describing differences in neuronal activity in indoor conditions with and without airflow.

We observed differences in gamma band activity between the two airflow conditions. The gamma amplitudes in the AC condition were higher than those in the RS condition, and this difference was identified in both the cooling and heating environments. Gamma synchronization has been shown to be associated with awareness and emotional content^20^, and especially unpleasant and aversive emotions^21–23^. In addition, a study of patients with an anxiety disorder indicated that they had higher gamma power in the temporoparietal regions than healthy participants^24^. We used analytic electrodes located at the same positions as those in a previous study^24^. In line with the previous study, the lower gamma activity may reflect less anxiety and/or less stress. Lower gamma amplitudes were observed in the RS condition, which may therefore indicate less anxiety and/or less stress in this condition. An explanation for why gamma activity is related to anxious state may lie in its relationship with the GABAergic network. Gamma oscillations can be generated in networks of GABAergic interneurons^25–27^. Several studies have reported that the GABAergic network is related to anxiety^28–30^. The subjective common scales from the PMV support this interpretation, as the scales are guaranteed to be in a comfortable state in the environment without airflow. Furthermore, we observed that PMV and PPD values correlate with gamma amplitude changes, as indicated by robust correlations and two-point slopes, indicating that gamma amplitudes are higher when uncomfortable feelings are increased. The correlations indicate that higher gamma activities reflect a state of anxiety in the airflow condition. Based on this perspective, we suggest that a mental state with less anxiety and/or less stress was induced by the airflow condition. This result likely indicates that airflow can influence feelings associated with environmental comfort. Gamma activity has also been associated with attentional processes^16, 17^, and the synchronization of gamma oscillations is related to sustained attention^16, 17, 31^ reflective of a heightened vigilance state. Therefore, our results may also indicate the presence of a more relaxed state characterised by lower vigilance under RS conditions.

Beta band amplitudes indicated a main effect of airflow, and the differences were observed near the motor cortex at C3 and C4. Several reports have confirmed that beta synchronization results from mental fatigue^18, 19^ in the primary motor cortex during tasks that require low mental load^19^, such as that used in our experiment. Differences in the beta band may therefore reflect changes in mental burden in the airflow condition, especially considering the concurrent changes in behavioural performance in the time-perception task. Participants overestimated the length of 1 minute more so in the RS condition than in the AC condition. That is, the relative speed of psychological time to the real time was faster in the RS condition. The change in time sensation under RS conditions may be caused by a decreased mental burden, such as decreased fatigue or boredom. From this perspective, the lower beta amplitudes in the RS condition may reflect a relaxing effect compared to the AC condition. Summarizing these results, we suggest that the airflow environment led to an increased mental burden.

The relationships between beta and the thermal scales (PMV or PPD) should be discussed. Although the PMV scores in the RS condition were within the comfort zone in both the cooling and heating experiments, we found significant correlations with PMV or PPD in the cooling experiment only, but not in the heating state. In the analysis of two-point slopes used to study relationships with PMV or PPD, we were unable to find any significant differences with chance levels. In this study, there was little evidence to support an involvement of beta in subjective feelings of thermal comfort. However, our discussion is not intended to reject the idea that the beta band was a candidate for a neurophysiological marker of airflow sensation, as there were significant differences in beta amplitudes between RS and AC, consistent with performance on the time-perception task.

Beta band activity in the motor area has been related to motor control and somatosensory sensation^32–37^. In our experiment, the participants were required to press a button with a finger in both conditions, so motor control is unlikely to underlie this difference. Somatosensory sensation seemed to be involved in the beta activity because of the impact of the airflow. In fact, previous studies have revealed that activities of the somatosensory area are reflected as beta rhythm suppression^38–41^. We did not observe such beta suppressions in the AC condition. Therefore, changes in the beta amplitudes are probably induced by mental fatigue, rather than somatosensory function.

The alpha band had significantly higher amplitudes in the AC condition than in the RC condition in only the heating experiment. Our data from the heating experiment are in agreement with those of previous studies, which have shown that attenuated alpha band amplitudes in the premotor cortex are associated with thermal sensations^42–44^. The attenuated alpha activity in the RS condition may thus reflect more sensitive thermal sensation.

The heterogeneous alpha patterns may reflect differences in the effectiveness of the airflow system to alter cooling and heating sensations. In the cooling environment, the AC and RS conditions did not show have differential effectiveness on cooling sensation, while the AC condition led to lower heat sensation than the RS system in the heating environment. The subjective assessments, however, indicate that airflow in a cooling environment is perceived as unacceptable, as it leads to local cooling of the skin, while appropriate air movement in a warm environment creates comfortable feelings^15^. Our results seem to contradict the above subjective perceptions of airflow. The observed heterogeneous patterns might be due to fundamental differences in the heat transfer systems, as we used far-infrared heating in the RS condition and convection heating in the AC condition.

In order to examine the neuronal mechanism underlying airflow sensation in an indoor environment, we compared the seasonal experiments in different airflow conditions using both electrophysiological and psychological approaches. In both the cooling and heating experiments, higher gamma and beta oscillation activities were observed as neurophysiological markers related to airflow sensation. These markers led to the identification of some candidates of brain-intrinsic factors related to feelings associated with different airflow conditions. This is the first report of neuronal evidence of airflow sensation in an indoor environment.

### Limitations

The sample size of this study was within a range of the common sense in human EEG studies though the size was smaller than that in healthy science areas including pharmacological studies. In this study, we removed some data as outliers because the current sample size was sensitive to the effect of abnormal values for parametric statistical analyses. The candidates of the outliers were selected automatically by a jackknife outlier analysis based on multivariate statistics. Although this data-driven method enabled to decide criteria of outliers without any arbitrary assumption, the method might not provide rationale to remove the outliers by itself because it could not clarify that the data categorized as outliers in this study were derived from noises or true outliers. In fact, the criteria were not identical between the cooling and heating experiments. This way of handling outliers might not be appropriate and could influence more or less the significance of the results. Nevertheless, there has been few studies about physiological responses under different thermal indoor environments, and any common consensus to eliminate outliers has not been established in the related studies. This study tried to investigate general features of physiological responses by eliminate outliers based on statistics though it let reduce the analytic data. To lead clearer conclusion, further investigations would be necessary with a larger sample size like pharmacological studies after a consensus of elimination data is established.

## Methods

### Participants

Twelve healthy volunteers participated in each experiment (cooling: 6 females and 6 males, 43–63 years old; heating: 6 females and 6 males, 44–66 years old) after providing written informed consent. The experiments were approved by the local ethics committee of Kyushu University. All methods were performed in accordance with the approved guidelines.

### Equipment

The experimental indoor condition with or without airflow was controlled using either a household air conditioner (AC; MSZ-GM560S, Mitsubishi Electric Corp., Tokyo, Japan) or a radiant cooling and heating system (RS; KFT System^TM^, KFT Co., Ltd., Fukuoka, Japan) (Fig. 1a) in a show house, which was used to mimic a typical modern living environment. Participants sat in a chair placed at the designated position (Fig. 1b). The environment was set to the same air temperature (median (interquartile range﻿)), AC: 25 (1.6) °C, RS: 24 (1.0) °C for the cooling experiment; AC: 26 (0.28) °C, RS: 26 (0.50) °C for the heating experiment) and the same relative humidity (AC: 64 (1.8)%, RS: 63 (2.8)% for the cooling experiment; AC: 24 (4.0)%, RS: 24 (3.4)% for the heating experiment) at the beginning of each experiment (Fig. 1b). The room temperature, relative humidity, and wind velocities were measured by an anemometer (AM-101, Kyoto Electronics Manufacturing Co. LTD., Kyoto, Japan), which was placed close to the participant (Fig. 1b). For the real-time monitoring by an experimenter, the room temperature and relative humidity were also measured by a digital hygrothermograph (RTR-53A, T&D Corp., Nagano, Japan), which was placed near the experimenter. The amount of clothing that each participant was wearing was standardised (Fig. 1c).

### Time-perception task

The task consisted of five sessions. In each session, the participants were required to press a button after counting 10 seconds without uttering words and with their eyes closed. In the first session, the participants pressed the button following time signals from a pure tone of 440 Hz every 1 second and of a pure tone of 880 Hz every 10 seconds. In the second through fifth sessions, the participants were required to estimate 10 seconds as exactly as possible and press the button without any signals as prompts. We measured the TTL (~60 seconds each session) that the participants perceived as a psychophysical factor. Before each session, the participants were asked to relax with their eyes closed; after each session, with their eyes opened. Each participant completed five sessions in each the two airflow conditions, AC and RS, in a random order.

### EEG recording

We recorded EEGs across 8 channels (Fz, Cz, Pz, Oz, C3, C4, T3, and T4) using gold active electrodes according to the international 10–20 system. The reference electrodes were placed on the tip of the nose, and the ground electrode was placed on Fpz. Additional two electrodes were placed on A1 and A2. The measurements were performed using a Polymate 2 (AP-216, Digitex Lab. Co. Ltd., Tokyo, Japan) before, during, and after the time-perception task. The EEG was amplified (with a 3-second time constant and low-pass 100 Hz filter) and digitalised at a sampling rate of 1000 Hz.

### EEG analysis

The measured EEG data were re-referenced to the averages of A1 and A2 and separated by the sessions of the task. The data were then transformed using discrete Fourier transform (DFT) with a rectangular window to obtain the DFT coefficients. DFT analysis was performed using the ‘fft.m’ function in MATLAB (MathWorks, Inc., Natick, USA). Each EEG data during each TTL (~60 seconds) was segmented into 20 time bins (~3 seconds). Amplitudes of every frequency bin calculated from the DFT coefficients were averaged within the following frequency bands: delta (0.5 ≤ *f* < 4 Hz), theta (4 ≤ *f* < 8 Hz), alpha (8 ≤ *f* < 14 Hz), beta (14 ≤ *f* < 30 Hz), and gamma (30 ≤ *f* < 55 Hz), where *f* indicated the frequency. The obtained amplitudes were averaged across 20 time bins in each frequency band. To assess relative changes compared to the first session, the mean amplitudes of each session were subtracted from those of the first session.

The subsequent analyses of gamma activities were restricted to the temporoparietal regions (Pz, T3, and T4) to assess the mental state of the participants^24^. Similarly, the analyses were restricted to two channels (C3 and C4) to study mental burden using beta activity^18, 19^ and to study differences in thermal sensation using alpha activity^42–44^. Theta activity was only analysed at Fz to assess concentration levels using “Fm-theta”, which is mainly observed in frontal regions^45^.

### Subjective thermal comfort

We calculated the PMV and PPD^5, 7^ scores based on environmental variables and the parameters measured during the experiments. PPD can be calculated from the PMV, as shown below^7^:

1 $${\rm{P}}{\rm{P}}{\rm{D}}=100-95\times \exp [-(0.03353\times {{\rm{P}}{\rm{M}}{\rm{V}}}^{4}+0.2179\times {{\rm{P}}{\rm{M}}{\rm{V}}}^{2})]$$

PMV and PPD were used to assess thermal comfort in both airflow conditions. All necessary variables used to obtain PMV were measured during the experiments. PMV, PPD, and related parameters were obtained using a PMV meter (AM-101, Kyoto Electronics Manufacturing Co. Ltd., Kyoto, Japan). For the PMV calculations, we set the amount of clothing to 0.7 (clo), and the metabolic rate to 1.0 (met) because the participants were seated.

### Thermography data recording

Thermography data used to assess skin temperature were measured five times per participant at the conclusion of each session in the cooling experiments, or twice per participant immediately before the first session and immediately after the fifth session in the heating experiments (Neo Thermo TVS-700, Nippon Avionics Co., Ltd., Tokyo, Japan). To assess the time-dependent changes in temperature in response to airflow, the first and last data points were analysed for each experiment. Although the timing of the measurements was different, we were able to compare the time-dependent changes in skin temperature in response to airflow in each experiment independently.

### Data removal process

The data removal process was as follows. First, we removed the data that were obtained with experimental errors from further analyses. Second, we selected the candidates of outliers by a jackknife outlier analysis based on a multivariate analysis using Mahalanobis distance by JMP^®^ 12 (SAS Institute Inc., Cary, NC, USA) for the remaining data. Third, we removed the data that were suspected to be with artifacts from the candidates.

The jackknife outlier analysis defined upper-control limits (UCL) distance to detect outliers based on the statistical alpha level (α = 0.05). The UCLs in EEG data in cooling and heating experiments were 4.0; the UCLs in behavioural data of the time-perception task in the cooling and heating experiments were 2.9; the UCLs for PMV or PPD in the cooling and heating experiments were 3.8. If a Mahalanobis distance of a data point was above UCL line, the participant including the data point was treated as candidate of outliers.

For statistical analyses of PMV and PPD, two sets of the data from the cooling experiment were removed because of the system trouble of the measurement apparatus for PMV and PPD (*i.e*., cooling: n = 10, heating: n = 12; Fig. 3). For statistical analyses of EEG data, in the cooling experiment, three participants were removed because of contaminating huge noise in the measurement apparatus for EEG; in the heating experiment, two participants who failed to complete the time-perception task were removed, and another participant was removed because of contaminating huge noise in the apparatus (*i.e*., cooling: n = 9, heating: n = 9; Figs 4–6). The exclusion criteria were that the mean amplitude of EEG delta band of each session >6.0 μV in cooling experiment or >60 μV in heating experiment. For statistical analyses of TTL from the time-perception task, in the cooling experiment, one participant was removed because of too much earlier responses; in the heating experiment, two participants were removed because they failed to complete the task as instructed, and another participant was removed because of too much later responses (*i.e*., cooling: n = 11, heating: n = 9; Fig. 7). The exclusion criteria were that the differential TTL from baseline of each session except for the 1st session < −15 seconds in cooling experiment or >95 seconds in heating experiment. For the other data, no outliers were found (*i.e*., cooling: n = 12, heating: n = 12; Figs 2 and 8).

### Statistics

Environmental parameters, such as room temperature, relative humidity, wind velocity, and summarising score (PMV and PPD) were analysed using non-parametric tests for comparisons between the conditions. Within-participant factors (EEG, behaviour, and skin temperature) were analysed using parametric tests. All statistical analyses were conducted by using JMP^®^ 12 (SAS Institute Inc., Cary, NC, USA).

The room temperature and relative humidity at the beginning of each experiment were analysed using a Wilcoxon test to compare between the AC and RS conditions. Wind velocities, PMV, and PPD values were averaged across the duration of each task through the five sessions, and a two-tailed Wilcoxon test was performed to compare between the AC and RS conditions.

Relative changes in EEG amplitude for each oscillation band between the first session and subsequent sessions were assessed using a two-way ANOVA (AC/RS × Sessions) for each electrode and in each season (cooling/heating). As mentioned above (see EEG analysis), we restricted the analysed electrodes before two-way ANOVA testing to reduce repetitions of post-hoc comparisons. Multiple comparisons were not performed because there were no significant interactions.

Relative changes in TTL between the first session and the subsequent sessions were assessed using a two-way ANOVA (AC/RS × Sessions) for each season (cooling/heating).

The thermography data were pooled. We performed K-means clustering to separate skin temperatures from background data in each condition and for each experiment (cluster number: 2). After separating the skin temperature data, they were averaged within subjects in each condition and experiment. A two-tailed Student’s *t*-test was performed to compare averaged skin temperatures between the first and fifth sessions, or the beginning and end of the experiment, in each condition.

### Data and code availability

The data and custom computer codes that support the findings of this study are available from the corresponding author upon reasonable request.

## Electronic supplementary material

Supplementary Information
