# Supplementary material for: Physiological activity in calm thermal indoor environments
Source: Sci Rep. 2017 Sep 14;7:11519. doi: 10.1038/s41598-017-11755-3 (PMC5599655; doi:10.1038/s41598-017-11755-3)
Supplement: Supplementary file 1 — Supplementary Information [file 41598_2017_11755_MOESM1_ESM.pdf]

Supplementary Information

Physiological activity in calm thermal indoor environments

Tsuyoshi Okamoto[1,2,3,*,+], Kaori Tamura[2,+], Naoyuki Miyamoto[4], Shogo Tanaka[4], and Takaharu Futaeda[4]

[1]Faculty of Medical Sciences, Kyushu University, 3-1-1 Maidashi, Higashi-ku, Fukuoka, 812-8582, Japan

[2]Faculty of Arts and Science, Kyushu University, 744 Motooka, Nishi-ku, Fukuoka, 819-0395, Japan

[3]Graduate School of Systems Life Sciences, Kyushu University, 744 Motooka, Nishi-ku, Fukuoka, 819-0395, Japan

[4]Anny Group, 6-3 Tenya-machi, Hakata-ku, Fukuoka, 812-0025, Japan

[+]these authors contributed equally to this work

[*]Correspondence and requests for materials should be addressed to T.O. (email: okamoto@artsci.kyushu-u.ac.jp).

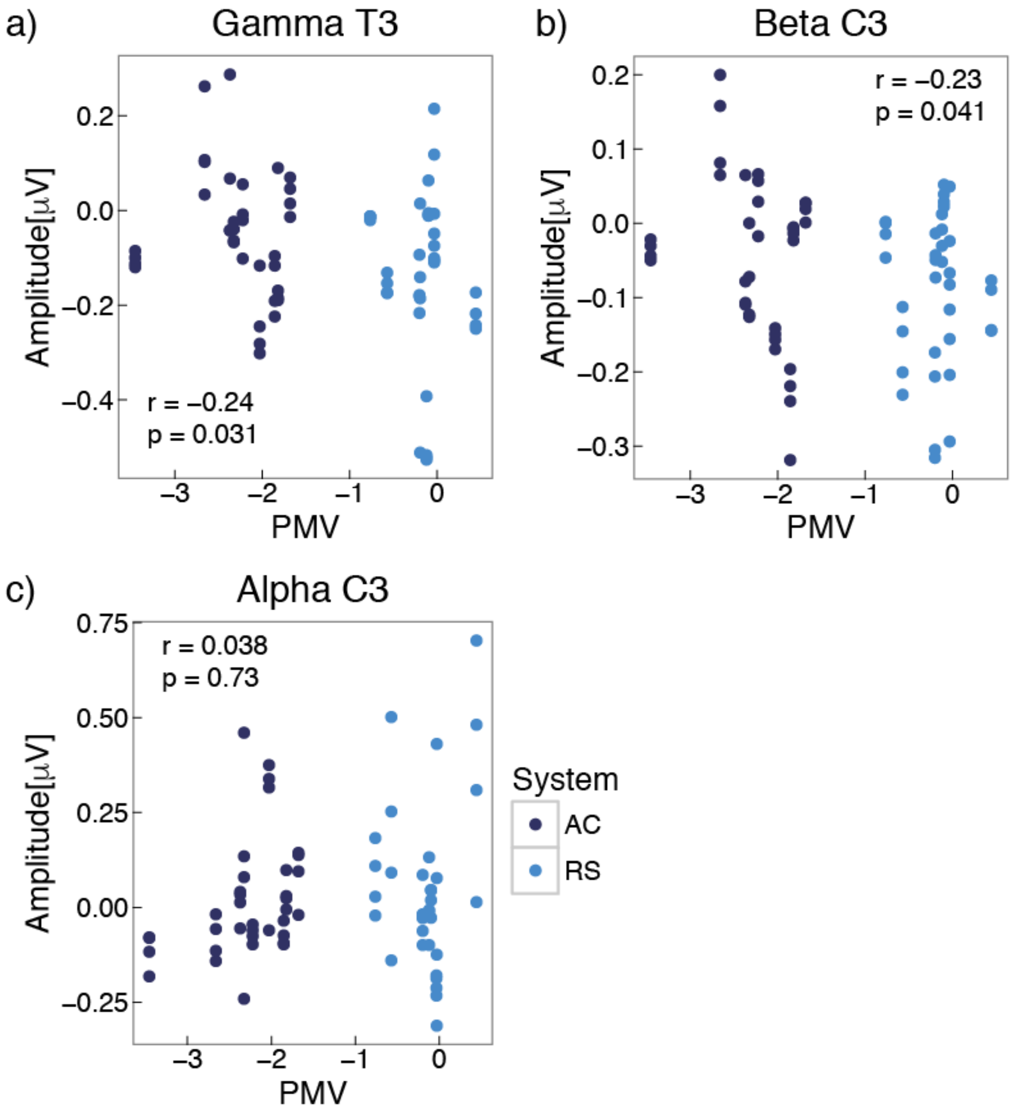

Figure S1. Correlations between PMV and the differential EEG amplitudes from the first session (2nd–5th sessions) in the cooling experiment. Outliers were removed using jackknife outlier analysis, *i.e.*, data from 9 participants were used for the correlation analysis. Each r and p value was calculated using robust correlation analysis.

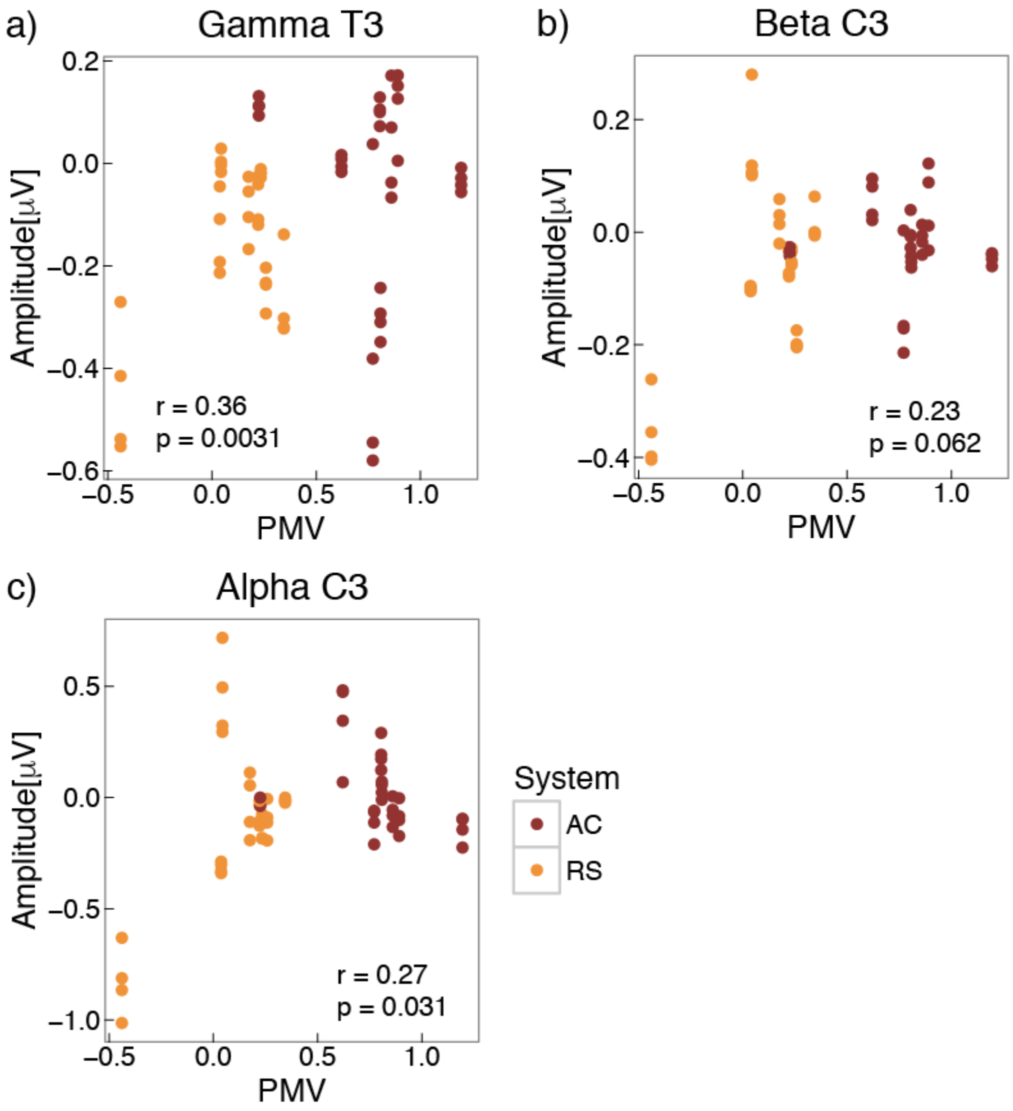

Figure S2. Correlations between PMV and the differential EEG amplitudes from the first session (2nd–5th sessions) in the heating experiment. Outliers were removed using jackknife outlier analysis, *i.e.*, data from 8 participants were used for the correlation analysis. Each r and each p was calculated using robust correlation analysis.

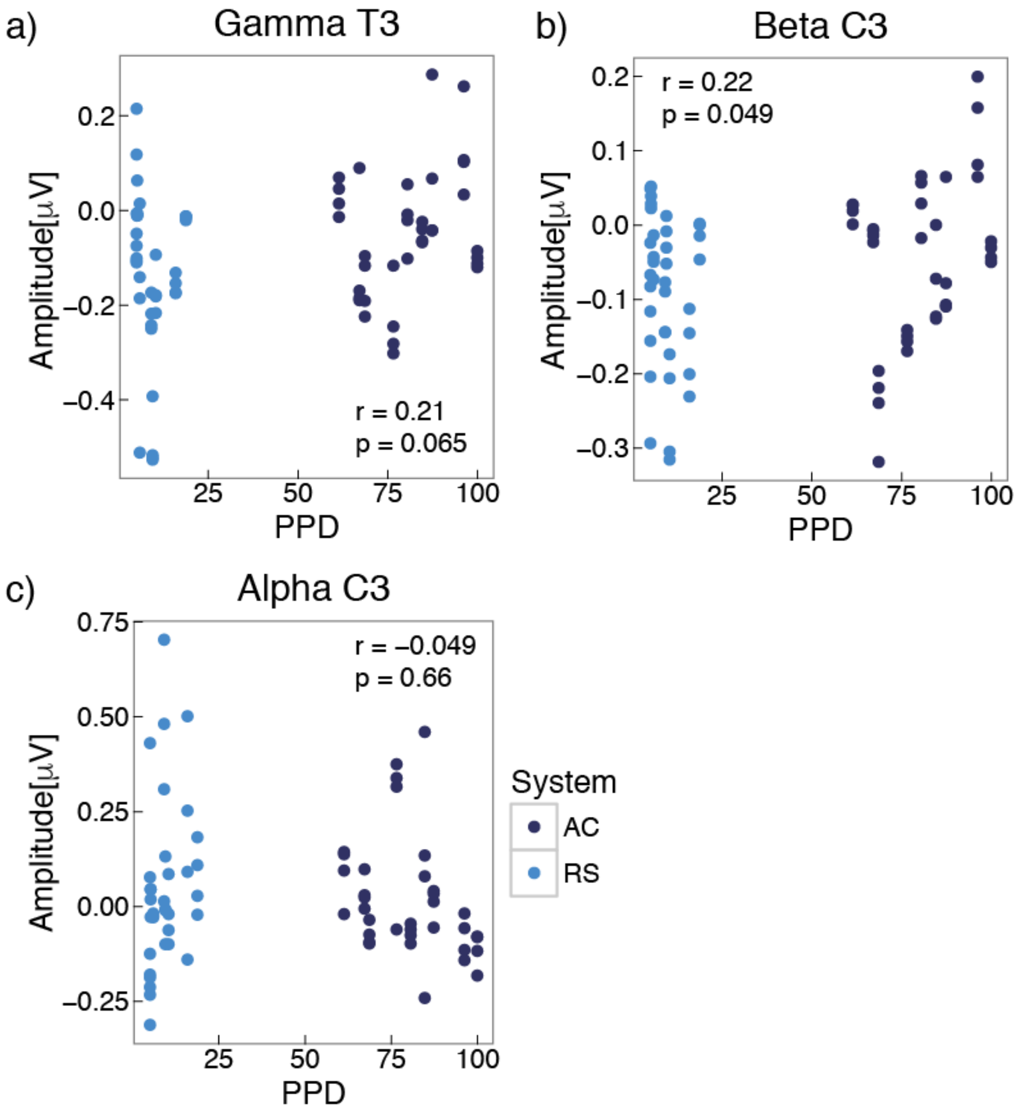

Figure S3. Correlations between PPD and the differential EEG amplitudes from the first session (session 2nd–5th) in the cooling experiment. Outliers were removed using jackknife outlier analysis, *i.e.*, data from 9 participants were used for the correlation analysis. Each r and p value was calculated using robust correlation analysis.

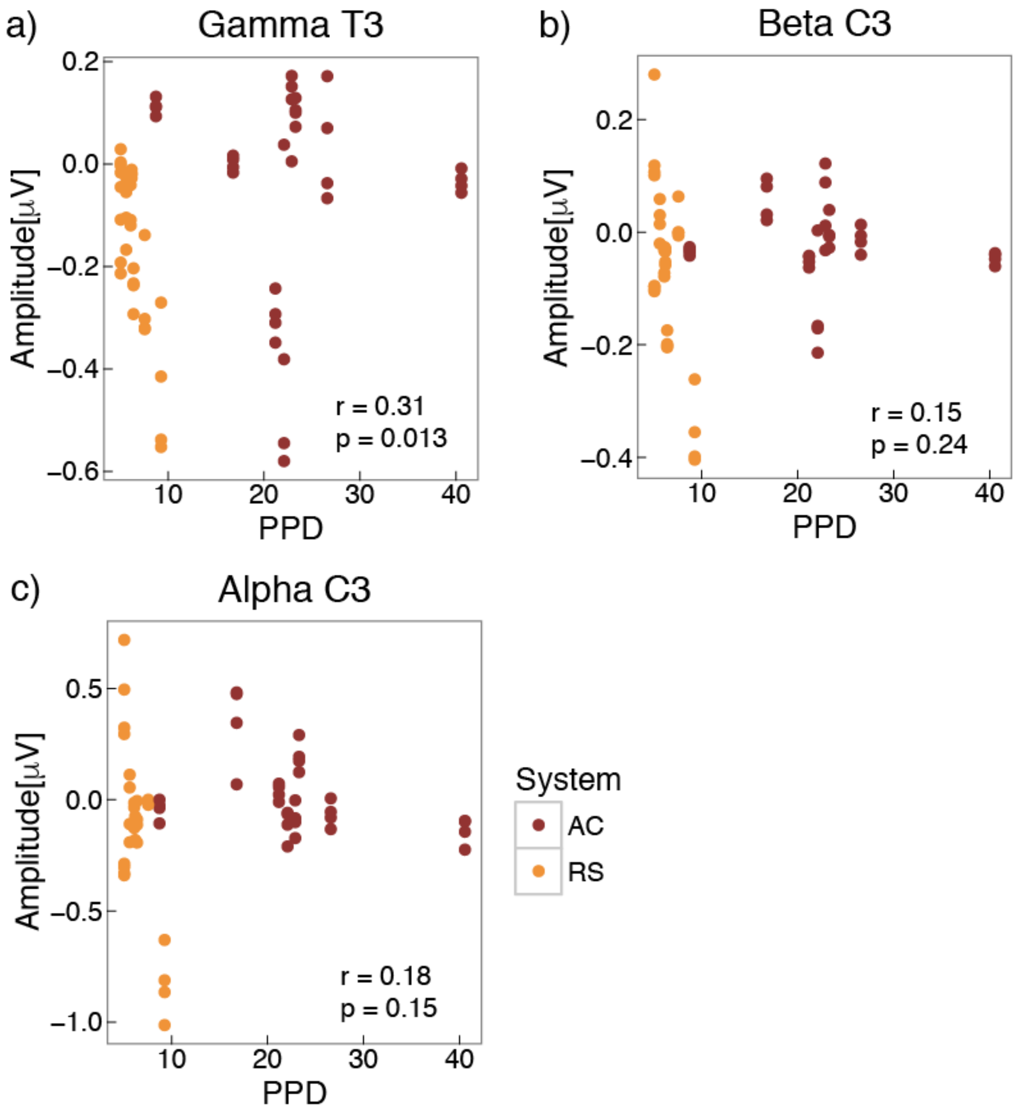

Figure S4. Correlations between PPD and the differential EEG amplitudes from the first session (session 2nd–5th) in the heating experiment. Outliers were removed using jackknife outlier analysis, *i.e.*, data from 8 participants were used for the correlation analysis. Each r and p value was calculated using robust correlation analysis.

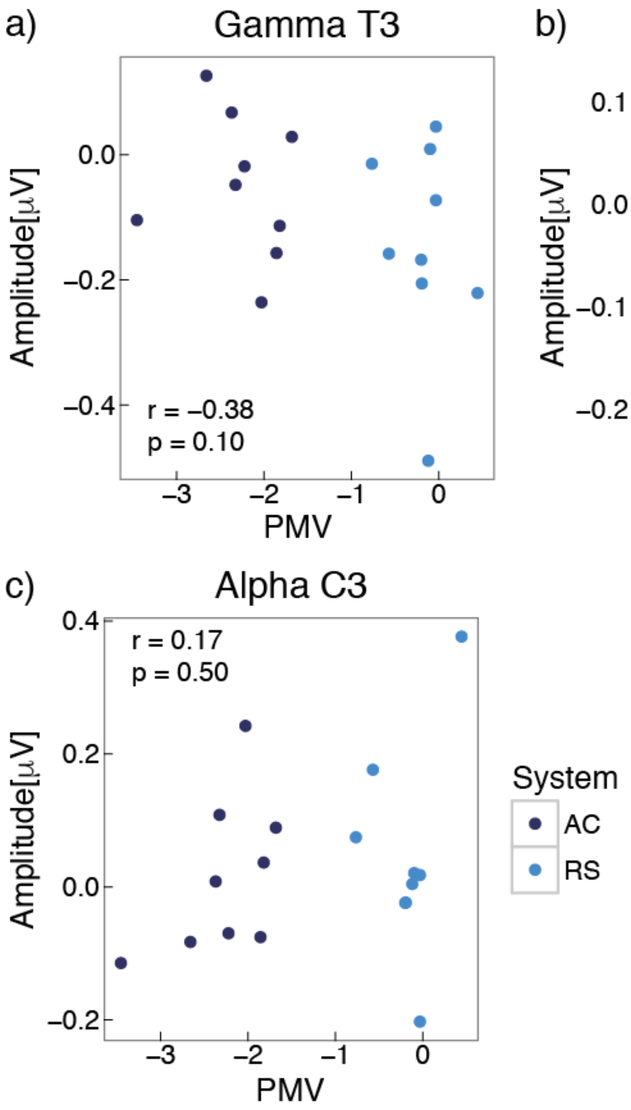

Figure S5. Correlations between averaged PMV and the EEG amplitudes across sessions (session 2nd–5th) in the cooling experiment. Outliers were removed using jackknife outlier analysis, *i.e.*, data from 9 participants were used for the correlation analysis. Each r and p value was calculated using robust correlation analysis.

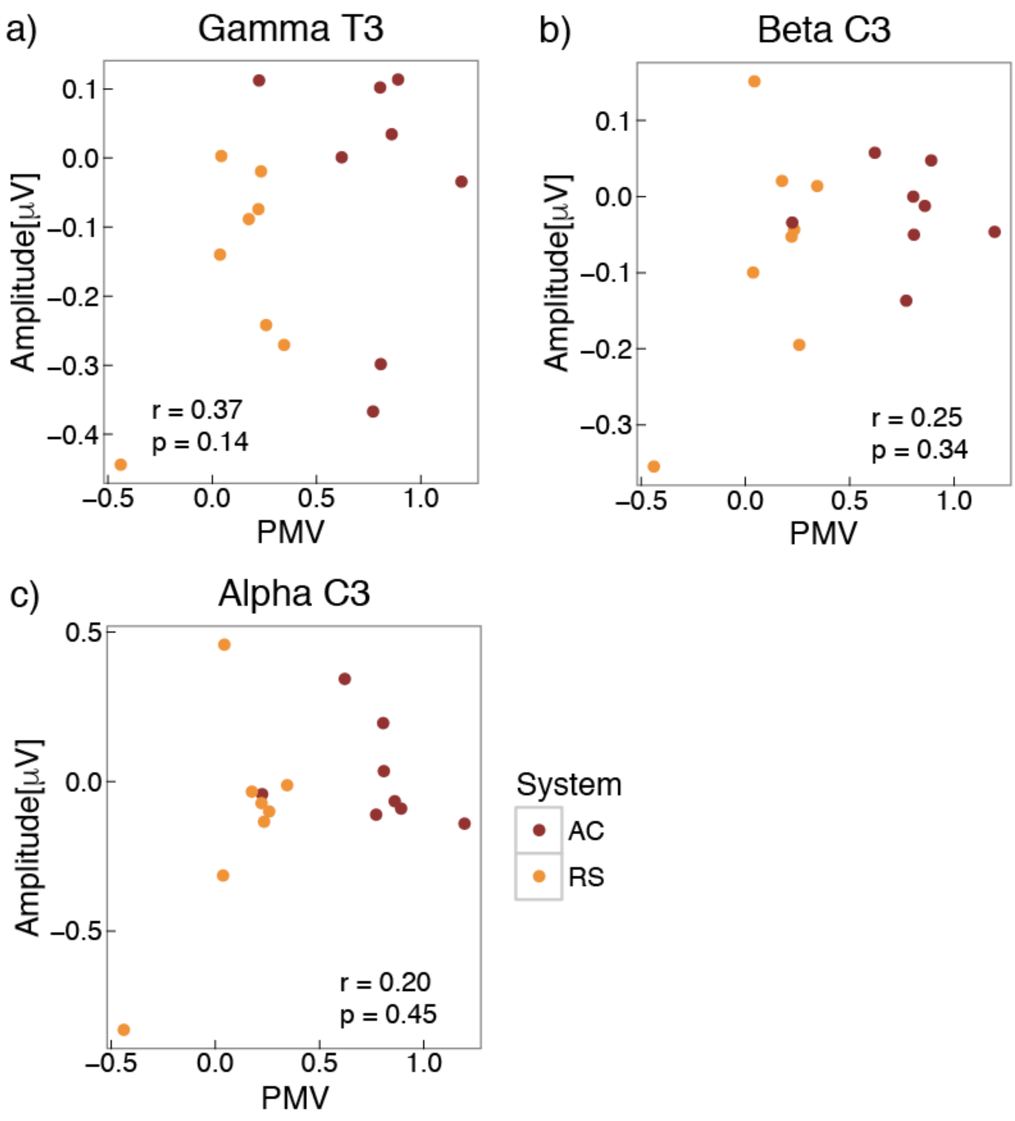

Figure S6 Correlations between averaged PMVs and EEG amplitudes across sessions (session 2nd–5th) in the heating experiment. Outliers were removed using jackknife outlier analysis, *i.e.*, data from 8 participants were used for the correlation analysis. Each r and p value was calculated using robust correlation analysis.

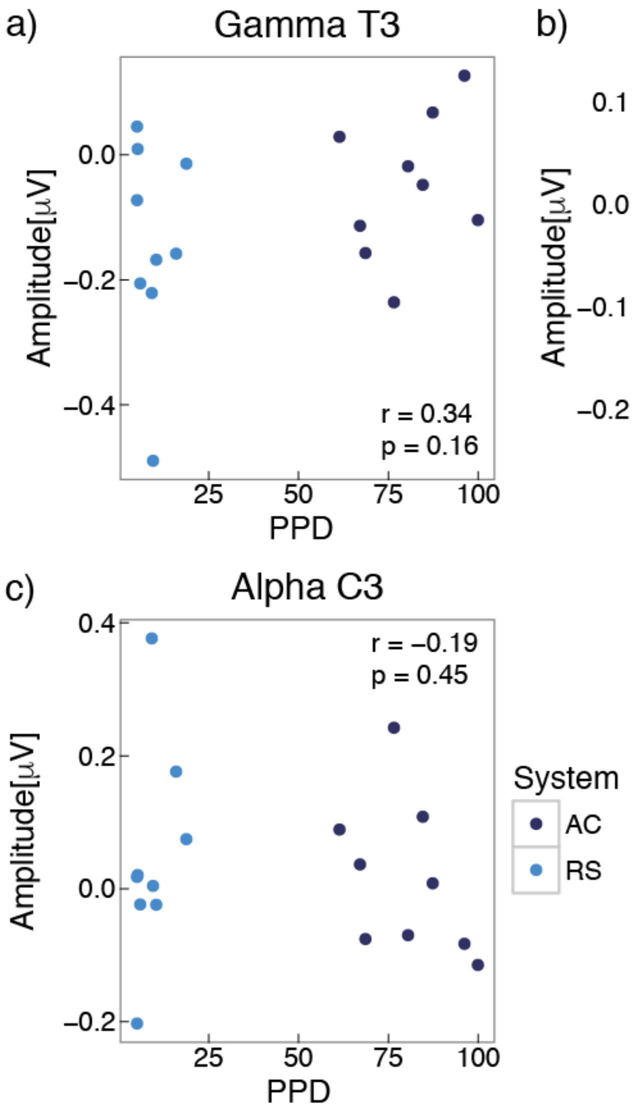

Figure S7. Correlations between averaged PPDs and EEG amplitudes across sessions (session 2nd–5th) in the cooling experiment. Outliers were removed using jackknife outlier analysis, *i.e.*, data from 9 participants were used for the correlation analysis. Each r and p value was calculated using robust correlation analysis.

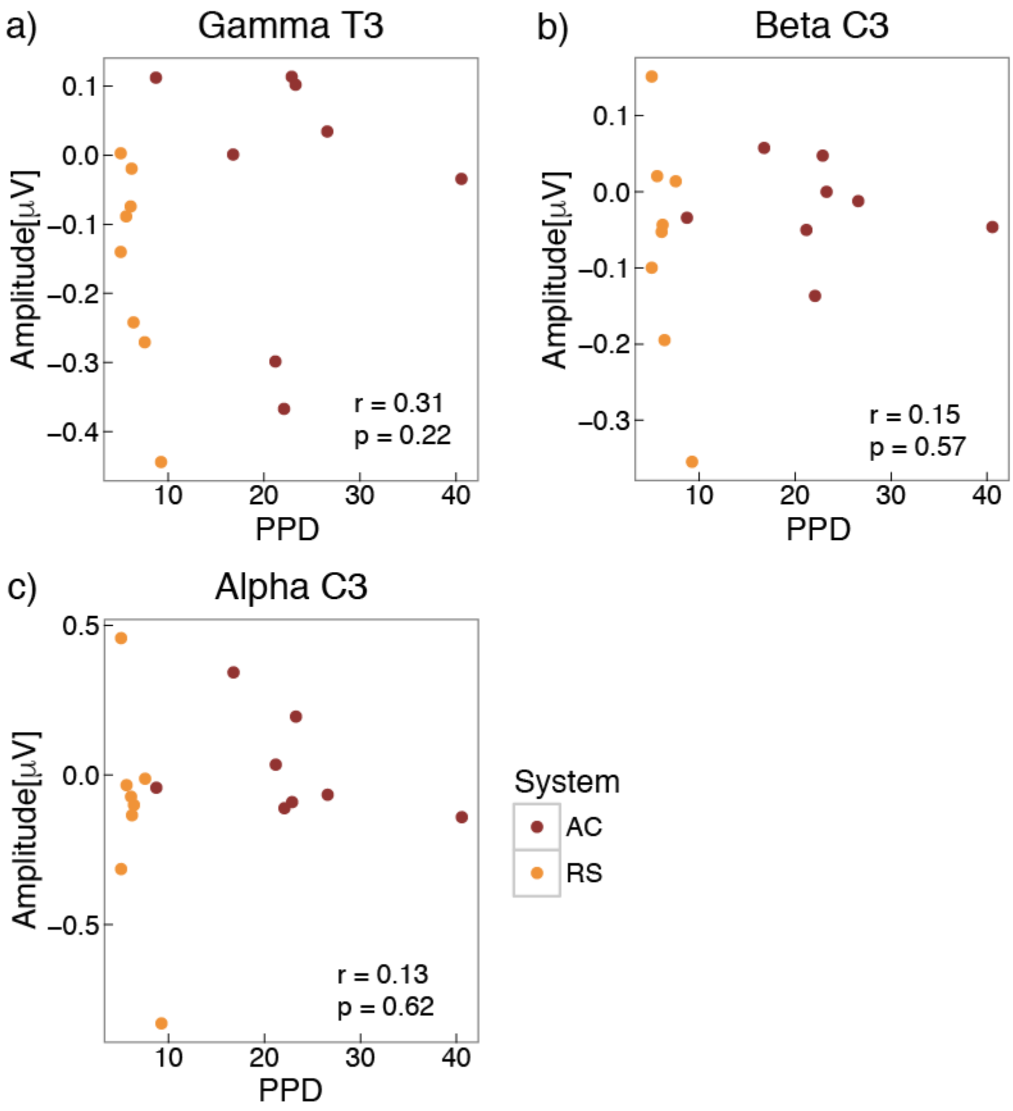

Figure S8. Correlations between averaged PPDs and EEG amplitudes across sessions (session 2nd–5th) in the heating experiment. Outliers were removed using jackknife outlier analysis, *i.e.*, data from 8 participants were used for the correlation analysis.

	Session 2	Session 3	Session 4	Session 5
Gamma (T3)	r = −0.36<br>p = 0.10	r = −0.20<br>p = 0.38	r = −0.32<br>p = 0.16	r = −0.027<br>p = 0.91
Beta (C3)	r = −0.18<br>p = 0.45	r = −0.25<br>p = 0.28	r = −0.18<br>p = 0.44	r = −0.22<br>p = 0.33
Alpha (C3)	r = 0.15<br>p = 0.51	r = −0.19<br>p = 0.42	r = 0.13<br>p = 0.57	r = 0.20<br>p = 0.38

Table S1. Correlation analysis between EEG and PMV in each session in the cooling experiment. Each r and p value was calculated using robust correlation analysis.

	Session 2	Session 3	Session 4	Session 5
Gamma (T3)	r = 0.35<br>p = 0.12	r = 0.18<br>p = 0.44	r = 0.29<br>p = 0.19	r = −0.0036<br>p = 0.99
Beta (C3)	r = 0.17<br>p = 0.46	r = 0.23<br>p = 0.31	r = 0.17<br>p = 0.45	r = 0.21<br>p = 0.36
Alpha (C3)	r = −0.14<br>p = 0.56	r = 0.20<br>p = 0.39	r = −0.099<br>p = 0.67	r = 0.20<br>p = 0.39

Table S2. Correlation analysis between EEG and PPD in each session in the cooling experiment. Each r and p value was calculated using robust correlation analysis.

	Session 2	Session 3	Session 4	Session 5
Gamma (T3)	r = 0.19<br>p = 0.46	r = 0.082<br>p = 0.76	r = 0.025<br>p = 0.92	r = 0.19<br>p = 0.47
Beta (C3)	r = 0.18<br>p = 0.49	r = 0.017<br>p = 0.95	r = 0.12<br>p = 0.64	r = 0.25<br>p = 0.33
Alpha (C3)	r = 0.38<br>p = 0.13	r = 0.29<br>p = 0.26	r = 0.44<br>p = 0.076	r = 0.24<br>p = 0.36

Table S3. Correlation analysis between EEG and PMV in each session in the heating experiment. Each r and p value was calculated using robust correlation analysis.

	Session 2	Session 3	Session 4	Session 5
Gamma (T3)	r = 0.25<br>p = 0.33	r = 0.099<br>p = 0.71	r = 0.076<br>p = 0.77	r = 0.24<br>p = 0.36
Beta (C3)	r = 0.051<br>p = 0.85	r = −0.10<br>p = 0.69	r = 0.058<br>p = 0.82	r = 0.19<br>p = 0.48
Alpha (C3)	r = 0.25<br>p = 0.34	r = 0.14<br>p = 0.58	r = 0.35<br>p = 0.17	r = 0.15<br>p = 0.57

Table S4. Correlation analysis between EEG and PPD in each session in the heating experiment. Each r and p value was calculated using robust correlation analysis.

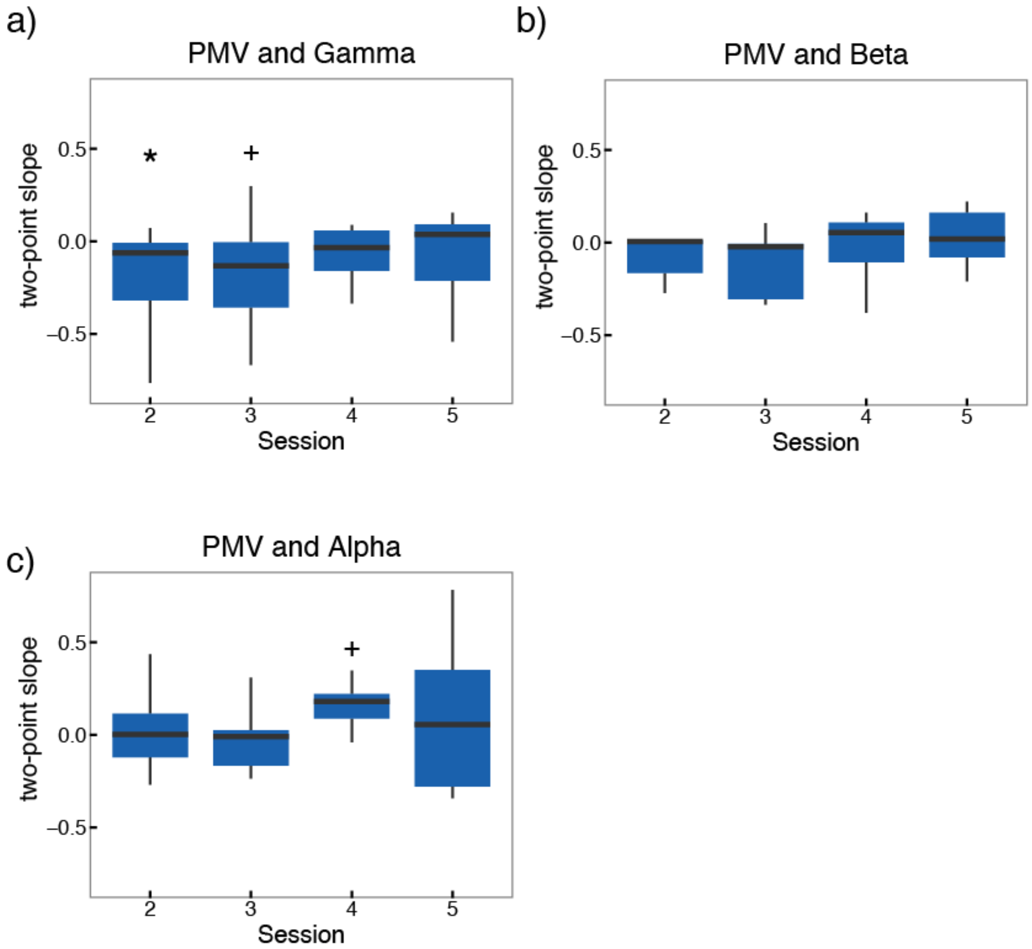

Figure S9. Two-point slopes of PMV in the cooling experiment in each session (Supplementary Methods). Asterisks indicate significance ($p < 0.05$) and daggers indicate marginal significance ($p < 0.10$) compared to zero.

	**Session 2**	**Session 3**	**Session 4**	**Session 5**
Gamma (T3)	Z = −16<br>p = 0.037	Z = −14<br>p = 0.065	Z = −7.5<br>p = 0.21	Z = −3.5<br>p = 0.37
Beta (C3)	Z = −4.5<br>p = 0.33	Z = −12<br>p = 0.10	Z = −1.5<br>p = 0.46	Z = −3.5<br>p = 0.37
Alpha (C3)	Z = 0.50<br>p = 0.50	Z = −4.5<br>p = 0.33	Z = 14<br>p = 0.065	Z = 4.5<br>p = 0.33

Table S5. Results of one-sample tests for two-point slopes of PMV in cooling. Each Z and p value was calculated using a one-sided Wilcoxon test between each median and zero.

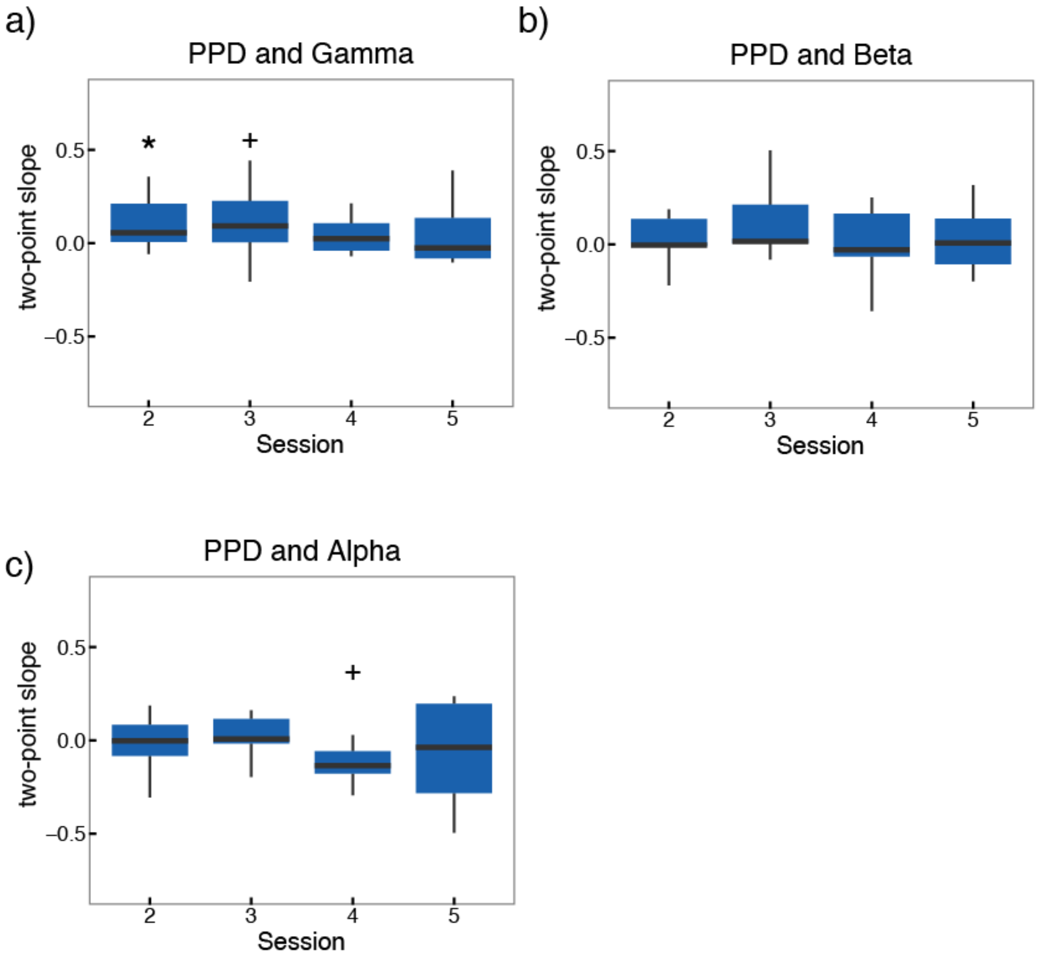

Figure S10. Two-point slope of PPD in the cooling experiment in each session (Supplementary Methods). Asterisks indicate significance ($p < 0.05$) and daggers indicate marginal significance ($p < 0.10$) compared to zero.

	**Session 2**	**Session 3**	**Session 4**	**Session 5**
Gamma (T3)	Z = 16<br>p = 0.037	Z = 14<br>p = 0.065	Z = 7.5<br>p = 0.21	Z = 3.5<br>p = 0.37
Beta (C3)	Z = 4.5<br>p = 0.33	Z = 12<br>p = 0.10	Z = 1.5<br>p = 0.46	Z = 3.5<br>p = 0.37
Alpha (C3)	Z= −1.5<br>p = 0.46	Z = 4.5<br>p = 0.33	Z = −14<br>p = 0.065	Z = −4.5<br>p = 0.33

Table S6. Results of one-sample tests for two-point slopes of PPD in cooling. Each Z and p value was calculated using a one-sided Wilcoxon test between each median and zero.

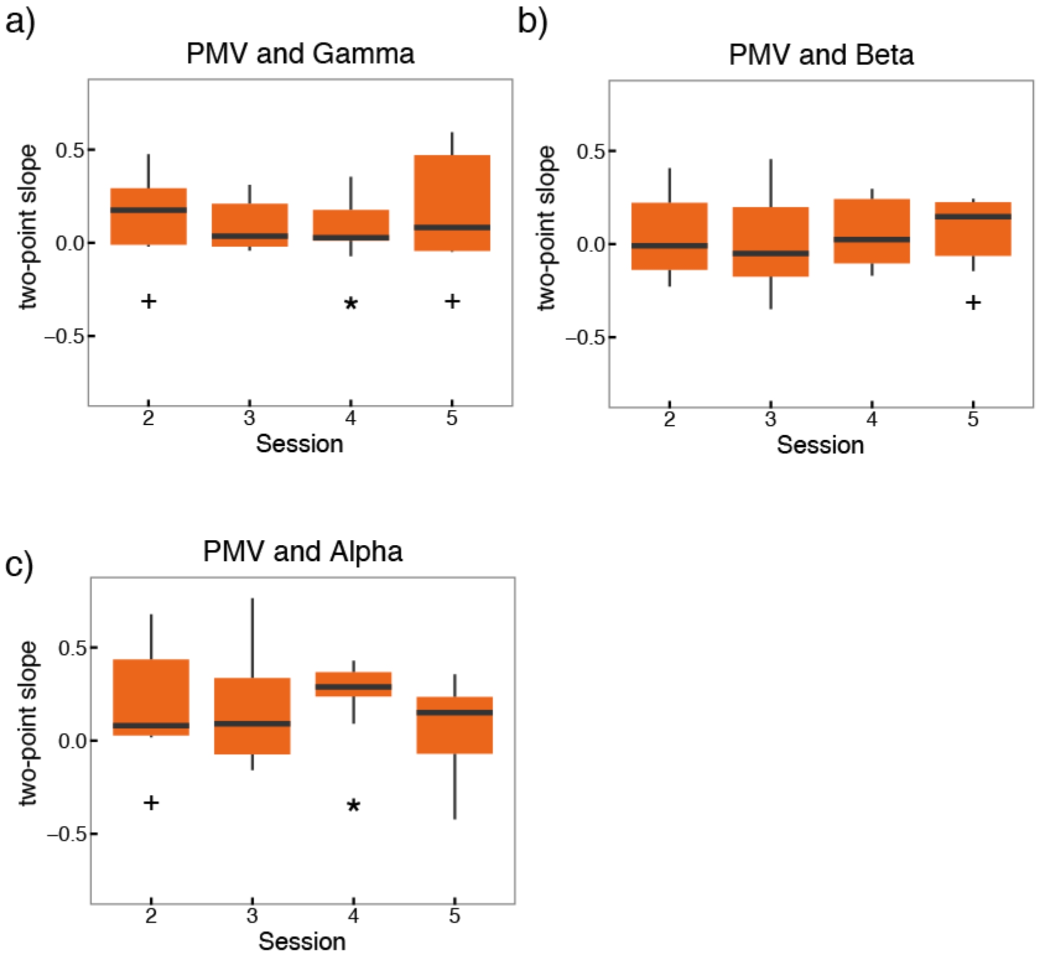

Figure S11. Two-point slopes of PMV in the heating experiment in each session (Supplementary Methods). Asterisks indicate significance ($p < 0.05$) and daggers indicate marginal significance ($p < 0.10$) compared to zero.

	**Session 2**	**Session 3**	**Session 4**	**Session 5**
Gamma (T3)	Z = 12<br>p = 0.055	Z = 9.0<br>p = 0.13	Z = 14<br>p = 0.027	Z = 12<br>p = 0.055
Beta (C3)	Z = −2.0<br>p = 0.42	Z = −4.0<br>p = 0.32	Z = 9.0<br>p = 0.13	Z = 10<br>p = 0.098
Alpha (C3)	Z = 11<br>p = 0.074	Z = 2.0<br>p = 0.42	Z = 18<br>p = 0.0039	Z = 8.0<br>p = 0.16

Table S7. Results of one-sample tests for two-point slopes of PMV in heating. Each Z and p value was calculated using a one-sided Wilcoxon test between each median and zero.

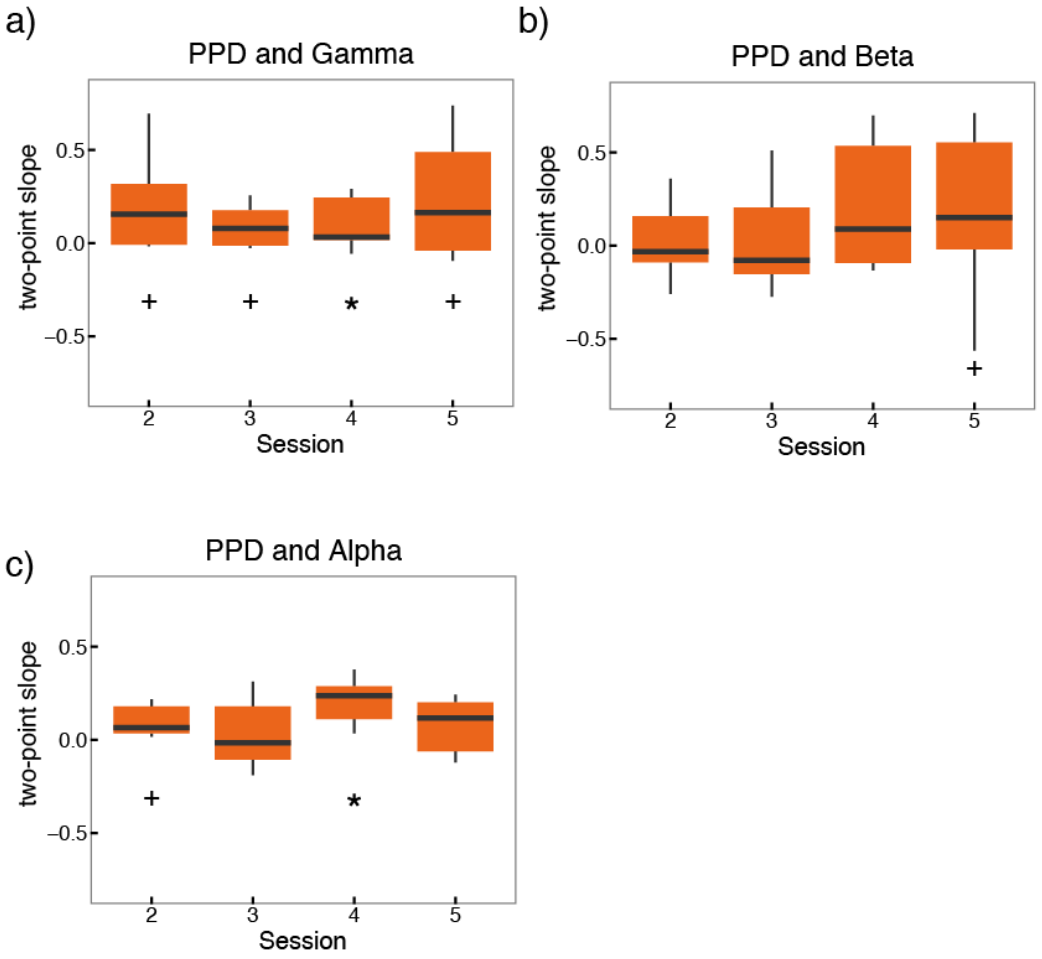

Figure S12. Two-point slopes of PPD in the heating experiment in each session (Supplementary Methods). Asterisks indicate significance ($p < 0.05$) and daggers indicate marginal significance ($p < 0.10$) compared to zero.

	**Session 2**	**Session 3**	**Session 4**	**Session 5**
Gamma (T3)	Z = 12<br>p = 0.055	Z = 10<br>p = 0.098	Z = 14<br>p = 0.027	Z = 12<br>p = 0.055
Beta (C3)	Z = 1.0<br>p = 0.47	Z = 1.0<br>p = 0.47	Z = 9.0<br>p = 0.13	Z = 10<br>p = 0.098
Alpha (C3)	Z= 11<br>p = 0.074	Z = 6.0<br>p = 0.23	Z = 18<br>p = 0.0039	Z = 8.0<br>p = 0.16

Table S8. Results of one-sample tests for two-point slopes of PMV in heating. Each Z and p value was calculated using a one-sided Wilcoxon test between each median and zero.

Supplementary Methods

To investigate the relationship between the thermal scale and EEG amplitude with independent observations, we introduced a new method to analyse a correlation in a simplified manner to calculate a new index, "two-point slope", which was defined as follows:

$$s = (y_2 - y_1)/(x_2 - x_1), \quad \text{(S1)}$$

$$[x_1,\ x_2] = [\text{nTSc(RS)},\ \text{nTSc(AC)}], \quad \text{(S2)}$$

$$[y_1,\ y_2] = [\text{nEEG(RS)},\ \text{nEEG(AC)}], \quad \text{(S3)}$$

where *s* is the two-point slope, nTSc is a normalized value across participants ranging from 0 to 1 for each thermal scale (PMV or PPD), and nEEG is a similar normalized amplitude for each EEG band (gamma, beta, or alpha). The value of *s* can be vary between −∞ and ∞ in principle, although its range is usually within ±1. The *s* value for each participant was obtained in each session. Median values of *s* that were significantly different from zero indicated that there was some relationship above chance level between the thermal scale and EEG amplitude as independent measurements (Figs. S9–12 and Tables S5–8).

The outlier of the participants were detected by a jackknife outlier analysis based on multivariate statistics. In the dataset combined EEG amplitudes and thermal scales (PMV and PPD), one participant in the heating experiment was further removed (cooling: n = 9, heating: n = 8).
